# The Development of a Novel Fiber-2 Subunit Vaccine against Fowl Adenovirus Serotype 4 Formulated with Oil Adjuvants

**DOI:** 10.3390/vaccines12030263

**Published:** 2024-03-01

**Authors:** Wenjian Liu, Meng Liu, Shuaiwen Wang, Zhihui Tang, Jiwen Liu, Suquan Song, Liping Yan

**Affiliations:** MOE Joint International Research Laboratory of Animal Health and Food Safety, Jiangsu Detection Center of Terrestrial Wildlife Disease, Institute of Immunology and College of Veterinary Medicine, Nanjing Agricultural University, Nanjing 210095, China; 2021107076@stu.njau.edu.cn (W.L.); 2019107078@stu.njau.edu.cn (M.L.); 2020807185@stu.njau.edu.cn (S.W.); tangzhihui@njau.edu.cn (Z.T.); 2020107098@stu.njau.edu.cn (J.L.); suquan.song@njau.edu.cn (S.S.)

**Keywords:** hepatitis-hydropericardium syndrome, fowl adenovirus serotype 4, prokaryotic expression, subunit vaccine, fiber-2, penton base

## Abstract

Hepatitis-hydropericardium syndrome (HHS), caused by fowl adenovirus serotype 4 (FAdV-4), has been widely spread across China, resulting in great financial losses in the poultry industry. Therefore, efficient vaccines against this disease urgently need to be developed. In our study, the fiber-2 and penton base proteins derived from the FAdV-4 JS strain were expressed in a prokaryotic system (*E. coli*) in a soluble form. Then, the efficacy of the two recombinant proteins formulated with cheap and widely used adjuvants (Marcol™ 52 white oil) were respectively tested, and the minimum immune doses and safety of the above proteins were also determined. It was indicated that the fiber-2 (20 µg/bird, 200 µg/bird) and penton base (200 µg/bird) could provide complete protection against the highly pathogenic FAdV-4 and suppress its replication and shedding. Unfortunately, only the fiber-2 protein could induce complete protection (10/10) at a low dose (10 µg/bird). In addition, we confirmed that the fiber-2 subunit vaccine formulated with oil adjuvants was safe for vaccinated chickens. Conclusively, all of our results suggest that we successfully prepared an efficient and cheap fiber-2 subunit vaccine with few side effects.

## 1. Introduction

Fowl adenovirus serotype 4 (FAdV-4), a member of the family *Adenoviridae* and genus *Aviadenovirus*, is an important causative agent of HHS characterized by pericardial effusion and hepatitis [1,2,3,4]. *Aviadenoviruses* are non-enveloped, double-stranded DNA viruses with icosahedral symmetry and a diameter of 70–90 nm [4]. Three main structural proteins, namely fiber, hexon, and penton base, constitute the capsid of FAdVs [5,6]. Hexon proteins play significant roles in the pathogenicity and replication of FAdV-4 [7,8,9,10,11]. The penton base is the vertex of the icosahedron on which two separate fiber proteins are anchored [4]. In addition, these two separate fiber proteins are essential for virus replication and participate in viral internalization [8,12,13,14,15].

FAdV-4, which mainly infects 3- to 5-week-old broilers [16,17], was first reported in Pakistan and later spread to other countries [18]. Since 2015, a novel highly pathogenic FAdV-4 has been spreading in many provinces of China, causing huge economic losses to the poultry industry [19,20,21,22,23]. The latest research shows that FAdV-4 could be isolated from wild birds and infect domestic mallard ducks, which has brought new threats to the poultry industry [24]. Vaccine development is particularly important for the prevention of this disease [25]. Subunit vaccines are easy to obtain and produce. More importantly, it is not only safe and efficient, but also pathogen-dissemination-free in vaccination, making them more attractive vaccine candidates [26,27]. However, there are only three commercial inactivated vaccines against FAdV-4 that are licensed in China [28]; the safe and effective commercial subunit vaccines are not available.

Fiber-2 and penton base proteins expressed in prokaryotic systems (*E. coli*) have potential as subunit vaccine candidates [29,30,31,32,33]. However, many scholars tend to use expensive adjuvants to enhance the immunogenicity of subunit vaccines [30,31,32,33,34]. Although the immunopotentiator effect of these adjuvants is undeniable, the high cost of them may limit the vaccine production.

Therefore, in the present study, we successfully expressed fiber-2 protein and penton base protein with high yield and good solubility. Based on this, we developed a subunit vaccine formulated with cheap and widely used oil adjuvants. It is indicated that subunit vaccines based on the fiber-2 protein could provide full protection against the highly pathogenic FAdV-4 with a low immune dose and few side effects.

## 2. Materials and Methods

### 2.1. Virus Propagation and DNA Extraction

The highly pathogenic FAdV-4 JS strain (GenBank accession number OR584077) was preserved in our lab. This virus was propagated on the Leghorn male hepatoma (LMH) cell line, and the median tissue culture infective dose (TCID_50_) was detected using endpoint titration. The viral DNA of the FAdV-4 JS strain was extracted from cell culture supernatant with Trelief™ Animal Genomic DNA Kit (TsingKe Biological Technology, Nanjing, China) according to the instructions.

### 2.2. Cloning, Protein Expression and Purification

The entire gene sequences for fiber-2 and penton base were amplified from viral DNA using the specific primers incorporated with 5′-terminal restriction sites *EcoR* I/*Xhol* I (Table 1). The PCR products were then cloned into the pET-28a vector to construct recombinant plasmids. The positive recombinant plasmids were identified by double digestion analysis, PCR, and sequencing.

The positive recombinant plasmids were transformed into *Escherichia coli* Rosetta (DE3) competent cells (Vazyme Biotech Co., Ltd, Nanjing, China) for the expression of fusion proteins. These transformed strains were cultured in LB medium containing 50 μg/mL kanamycin at 37 °C until the OD_600_ reached 0.4–0.6. Then 0.5 mM isopropyl-β-D-thiogalactopyranoside (IPTG) was added to induce the expression of the fusion proteins and further cultured at 16 °C overnight. Bacterial cells were harvested by centrifugation at 12,000 rpm for 10 min at 4 °C and then resuspended with lysis buffer (20 mM imidazole, 500 mM NaCl, 20 mM NaH_2_PO_4_, pH 8.0). Sonication was performed to disrupt the collected bacterial cells. The supernatant and pellet were separated by centrifugation, and then, all the proteins were purified on affinity chromatography columns with Ni-NTA agarose (QIAGEN, Duesseldorf, Germany) according to the instructions. Purified recombinant proteins were concentrated through ultrafiltration with Amicon^®^ Ultra 15 mL Centrifugal Filters (Merck Millipore, Billerica, MA, USA) and quantified using the BCA protein Quantification Kit (Vazyme Biotech Co., Ltd, Nanjing, China).

SDS-PAGE and Western blot analysis were then performed to demonstrate the expression level and purity. After electrophoresis, the gel was stained with Coomassie Brilliant Blue Staining Solution. The other gel was transferred onto nitrocellulose membrane, and the membrane was incubated with anti-His-tag antibody (Biodragon Immunotechnologies Co., Ltd, Beijing, China) (1:2000), chicken serum against penton base (1:2000), and anti-fiber-2 monoclonal antibody. The goat anti-mouse antibody (KPL, Gaithersburg, MD, USA), or goat anti-chicken antibody (KPL, Gaithersburg, MD, USA), was used as a secondary antibody. Antibody response bands were detected by enhanced chemiluminescence catalyzed by ECL luminous fluid (Vazyme Biotech Co., Ltd, Nanjing, China).

### 2.3. Immunization and Challenge

The purified recombinant proteins were emulsified in a ratio of 1:2 (*v*:*v*) with Marcol™ 52 white oil (Exxon Mobil Corporation, Irving, TX, USA). A total of 120 seven-day-old SPF chickens were randomly assigned to four vaccinated groups, one challenged control group, and one negative control group (n = 20 per group). The vaccinated chickens (Groups I–IV) were subcutaneously injected with subunit vaccines (300 μL at one site) on the nape. Group V was challenged but not vaccinated. Chickens Group VI were subcutaneously injected with PBS (300 μL at one site) on the nape. The six groups were housed individually in different negative-pressure isolators.

Three weeks after immunization, all groups except group VI were challenged with 2 × 10^6.5^ TCID_50_ of FAdV-4 JS strain via intramuscular injection. A total of 20 chickens in each group were further divided into two groups with 10 chickens each, based on numbers marked on their feet. All chickens in one group were observed daily for clinical symptoms and the mortality rates were calculated at the end of the trial. In the other group, three chickens were euthanized and necropsied at 1, 3, and 5 days post challenge (dpc), respectively. All remaining chickens were euthanized at the trial termination at 14 dpc.

To further explore the protective efficiency of fiber-2 protein and penton base protein, 90 seven-day-old SPF chickens were divided into nine groups (n = 10 per group), which included seven vaccinated groups (groups A–G), one challenged control group (group H), and one negative control group (group I). The chickens in groups A–D were inoculated with 40 μg, 20 μg, 10μg, and 5 μg of recombinant fiber-2 protein, respectively. The chickens in groups E–G were inoculated with 100 μg, 50 μg, and 25 μg of recombinant penton base protein, respectively. Then, all the chickens except for those in group I were challenged with 2.0 × 10^6.5^ TCID_50_ of FAdV-4 JS strain at 21 days post immunization (dpi) as mentioned above. The chickens were observed daily for clinical symptoms and survival rates were calculated at the end of the experiment. All groups were housed individually in different negative-pressure isolators. All remaining chickens were euthanized at the trial termination at 14 dpc.

To confirm the safety of the subunit vaccine, the purified recombinant fiber-2 proteins with a concentration of 0.1 mg/mL were emulsified in a ratio of 1:2 (*v*:*v*) with Marcol™ 52 white oil. Then, a total of 20 seven-day-old SPF chickens were randomly assigned to four groups (groups 1–4, n = 5 per group). Each chicken in Group 1, the conventional dose injection group, was subcutaneously injected with a dose of 300 μL (300 μL at one site). Each chicken in Group 2, the continuous conventional dose injection group, was subcutaneously primarily injected with a dose of 300 μL (300 μL at one site) and boosted with same dose at 14 dpi. Each chicken in Group 3, the over-dose injection group, was subcutaneously injected with a dose of 1000 μL (1000 μL at one site). Group 4 served as the negative control group. The chickens were observed daily, and the body weight of all chickens was measured every 3 days until 21 dpi. All chickens were euthanized and necropsied to observe the vaccination site at trial termination at 21 dpi. 

### 2.4. Enzyme-Linked Immunosorbent Assay (ELISA)

Blood was collected from chickens in each group to prepare serum samples at 0, 7, 14, and 21 dpi. The level of protein-specific antibodies was determined by ELISA. The envelop antigens, purified fiber-2, and penton base proteins, were prepared at a concentration of 0.2 μg/well and coated with 96-well ELISA plates (Jet Bio-Filtration Co., Ltd, Guangzhou, China), respectively. Sera collected from experimental chickens vaccinated with the matching antigens were diluted at a ratio of 1:200, added to each well and incubated at 37 °C for 1 h. The goat anti-chicken IgG at a dilution of 1:5000 was used as a secondary antibody and incubation was performed at 37 °C for 30 min. Following incubation with the chromogenic solution, the reaction was terminated with 0.5 M sulfuric acid and then the OD_450_ of each well was detected. The cut-off value was calculated based on the values of negative sera; it is 2.1 times the OD_450_ values of the negative sera. Each test was conducted three times [34].

### 2.5. Histopathology and Immunohistochemistry Analyses

Tissues of the heart, liver, and spleen from chickens euthanized at 3 dpc in each group were fixed in 4% paraformaldehyde solution for 72 h, embedded in paraffin wax, sliced, and stained with hematoxylin and eosin (H&E). Furthermore, the slices were stained by immunohistochemistry to detect the viral antigen contents in the heart, liver, and spleen tissues. Home-made mouse anti-FAdV-4 monoclonal antibody against fiber-2 and goat anti-mouse IgG antibody were incubated with tissue sections for 1 h in turn. The sections were dehydrated and mounted after counterstaining with hematoxylin. 

### 2.6. Real-Time PCR

Three chickens were randomly selected from each group and euthanized to collect tissue samples of heart, liver, spleen, lung, and kidney at 1, 3, and 5 dpc. Cloacal swabs from each group were collected daily for 5 dpc. Total DNA was extracted from tissue samples and cloacal swabs with a Trelief™ Animal Genomic DNA Kit (TsingKe Biological Technology, Naning, China), and then SYBR Green I quantitative PCR assay (Vazyme Biotech Co., Ltd, Nanjing, China) was used to quantify the viral DNA in different tissues. The primers of FAdV-4 ORF14 gene used in this study are shown in Table 1 [8]. The reactions were conducted with the cycling program based on instructions. The standard curve was produced by a recombinant plasmid embracing the 136bp fragment of the FAdV-4 ORF14 gene from 10^1^ to 10^7^ copies/μL.

### 2.7. Statistical Analysis

Statistical analysis was performed using the GraphPad Prism 9.5.1 software package. Normality and lognormality tests should be first preformed to assess the distribution of the data. All data should conform to the normal distribution before preforming two-way analysis of variance (ANOVA). To analyze the viral loads and antibody levels, comparisons were made between the vaccinated and challenge control groups. Comparisons were also made between the vaccinated and negative control groups to analyze the body weight gain of the vaccinated chickens. Significant differences were considered as * *p* < 0.05, ** *p* < 0.01, *** *p* < 0.001, or **** *p* <0.0001.

## 3. Results

### 3.1. Construction of Expression Vectors

The fiber-2 and penton base genes were successfully amplified from the genomic DNA of the FAdV-4 JS strain by PCR using the primers’ pairs incorporated with the 5′-terminal restriction sites *EcoR* I/*Xhol* I. The expected amplified products of 1440 bp and 1578 bp were verified by agarose gel electrophoresis and then cloned into a pET-28a vector. The presence of a foreign gene in the recombinant plasmid was confirmed by PCR. A DNA sequencing analysis demonstrated that the recombinant plasmids were constructed correctly.

### 3.2. Expression and Purification of Recombinant Proteins

**Figure 1:**
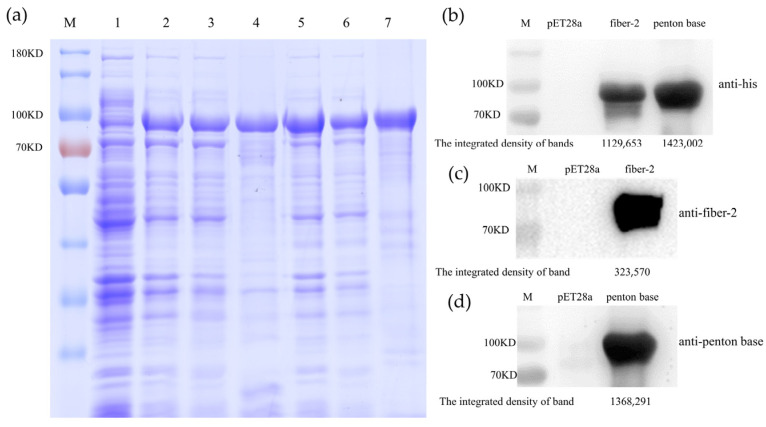
The expression verification of fusion recombinant fiber-2 and penton base protein. SDS-PAGE analysis of soluble and pellet of *E. coli* expressing penton base and fiber-2 proteins (**a**). Lane M, protein ladder. Lane 1, *E. coli* Rosetta (DE3) containing pET-28a with IPTG induction. Lane 2, total extracts of *E. coli* Rosetta (DE3) containing pET-28a-fiber-2. Lane 3, soluble extracts of *E. coli* Rosetta (DE3) containing pET-28a-fiber-2. Lane 4, purified recombinant fiber-2 protein. Lane 5, total extracts of *E. coli* Rosetta (DE3) containing pET-28a-penton base. Lane 6, soluble extracts of *E. coli* Rosetta (DE3) containing pET-28a-penton base. Lane 7, purified recombinant penton base protein. Western blot analysis of the two purified recombinant proteins probed by anti-histamine monoclonal antibody (**b**). Western blot analysis of the purified fiber-2 protein probed by anti-Fiber-2 monoclonal antibody (**c**). Western blot analysis of the purified penton base protein probed by anti-penton base positive chicken serum (**d**). The integrated density of bands was calculated by using the Image J 1.44p software.

SDS-PAGE and Western blot were used to identify the expression levels and purity of the two recombinant proteins. The results of SDS-PAGE demonstrated that recombinant proteins were successfully expressed in soluble form at approximate sizes of 89 KD (fiber-2) and 94 KD (penton base), respectively (Figure 1a). The expression rates of the fiber-2 and penton base proteins in supernatant were 32.5% and 47.5%, respectively, as calculated using the BandScan V5.0 software. Western blot revealed that the recombinant his-tagged target proteins can react with the anti-his monoclonal antibodies (Figure 1b). Fiber-2 protein could react with the anti-his monoclonal antibodies (Figure 1c). Penton base protein could react with the chicken polyclonal antibodies (Figure 1d). After purification by Ni-NTA chromatography and ultrafiltration concentration, the concentration of fiber-2 proteins reached 1.5 mg/mL and that of the penton base proteins reached 2.3 mg/mL. In addition, the yield of purified recombinant proteins obtained per liter LB medium could reach almost 170 mg (fiber-2) and 150 mg (penton base).

### 3.3. Challenge Protection Test and Detection of Antibody Titers

**Figure 2:**
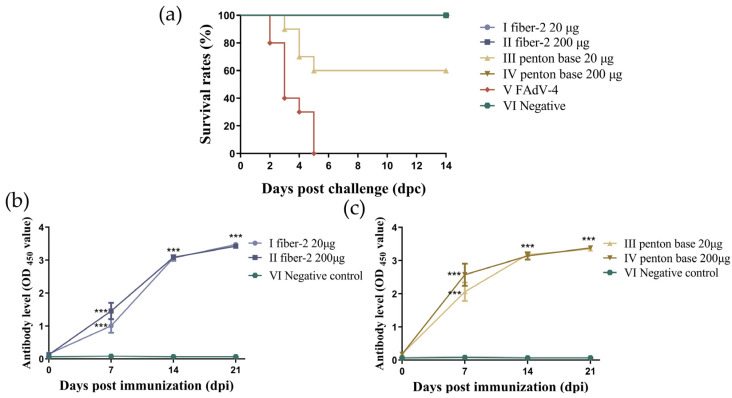
Challenge protection test and detection of antibodies’ titer. Survival curves of all chickens in challenge protection test following challenge with 2 × 10^6.5^ TCID_50_ highly pathogenic FAdV-4 JS strain at 21 dpi (**a**). The mortality rate was recorded for 14 dpc. The average OD_450_ values of chickens at 0, 7, 14, 21 dpi in vaccinated groups and negative control group. The statistical significance between vaccinated groups and negative control group was considered as *** *p* < 0.001 (**b**,**c**).

To assess the protection efficiency of two recombinant proteins, we conducted a challenge protection test. The results indicate that both doses of fiber-2 (20 µg/bird and 200 µg/bird) could induce complete protection (10/10) at 21 dpi. However, penton base can only confer complete protection (10/10) at a higher dose (200 µg/bird) (Figure 2a).

Specific antibodies against recombinant proteins were detected in the sera of the immunized chickens via an indirect ELISA (Figure 2b,c). Serum antibody responses before immunization were not detected in any chickens before immunization, and the cut-off value was 0.3423. The average OD_450_ values of the sera from the chickens vaccinated with two doses of fiber-2 and penton base proteins were 1.006, 1.458, 2.059, and 2.576, respectively, at 7 dpi, which were significantly higher than those of the negative control group (*p* < 0.001). The antibodies were detected in all immunized chickens except for one chicken immunized with 20 μg of fiber-2 protein at 7 dpi. At 14 dpi, the average OD_450_ values of each immunization group were 3.067, 3.085, 3.180, and 3.139, respectively, which was remarkably higher than those at 7 dpi. The antibody titers of the vaccinated chickens were the highest at 21 dpi, and the values were 3.475, 3.423, 3.350, and 3.382, respectively. These results demonstrated that both fiber-2 and penton base protein induced effective immune responses, causing a gradual increase in serum antibody level.

### 3.4. Viral Loads in Target Tissues and Cloacal Swabs

**Figure 3:**
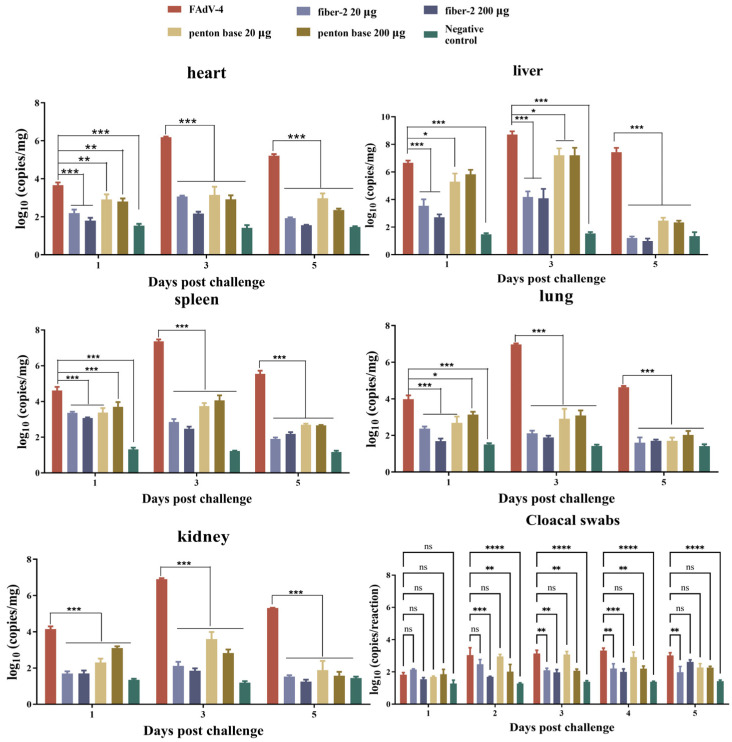
Viral loads in target organs and cloacal swabs of chickens in challenge protection test. Viral loads in different tissues were determined by a real-time RCR using the primers of FAdV-4 ORF14 gene. Mean value and standard deviation of viral DNA were considered as log_10_ (copies/mg). *p* values were calculated with two-way ANOVA. * *p* < 0.05, ** *p* < 0.01, *** *p* < 0.001, or **** *p* < 0.0001.

Samples of heart, liver, spleen, lung, and kidney tissues and cloacal swabs were taken from chickens in each group at designated time points and viral genomic copy numbers in each tissue were detected by quantitative real-time PCR. As shown in Figure 3, viruses were detected with high titers in the tissues from the challenged control group at 1, 3, and 5 dpc and reached maximum genomic copy numbers at 3 dpc. Among these tissues, the viral load of liver is the highest, followed by the spleen, lung, kidney, and heart. The viral loads in tissues of chickens from the negative control group served as the background, and the viral loads in the tissues of chickens from groups I–IV were significantly lower than those of the challenge control group. The viral loads of cloacal swabs from groups II and IV were significantly lower than those from the challenged control group at 2–4 dpc. And the viral loads of cloacal swabs in group I were significantly lower than those in the challenged control group at 3–5 dpc. However, the viral loads of the cloacal swabs from all immunization groups were higher than those of the negative control group. The results demonstrate that the fiber-2 and penton base protein could effectively prevent viral replication in these target tissues but may not completely reduce viral excretion.

### 3.5. Gross Lesions, Histopathology and Immunohistochemistry

**Figure 4:**
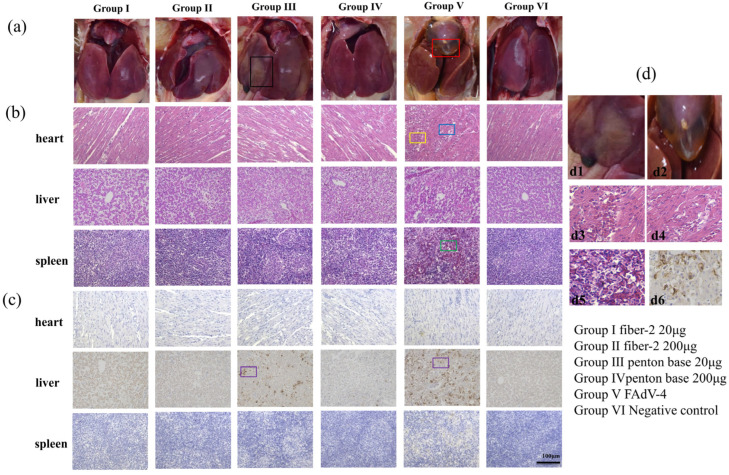
Observation of gross lesions (**a**), histological lesions (**b**), detection of viral antigens (**c**), in different tissues from chickens in challenge protection test at 3 dpc. The partial enlarged view of the highlighted sections of black, red, yellow, green, and purple boxes (**d**). The main gross lesions in challenge control group included yellow-stained livers accompanied with necrosis and bleeding (black box, (**d1**)) and pericardial effusion (red box, (**d2**)). Histological lesions such as myocardial interstitial hyperemia (yellow box, (**d3**)), myocardial fiber fracture (blue box, (**d4**)), and necrosis of the lymphocytes (green box, (**d5**)) can be observed. Many viral particles in the liver slices of chickens in challenge control group can be detected and a few viral particles were observed in the liver sections of the chickens vaccinated with 20 μg penton base protein (purple box, (**d6**)).

As early as 2 dpc, chickens in the challenged control group began to show typical HHS-related clinical symptoms, such as depression, anorexia, and ruffled feathers. The deaths occurred between 2 and 5 dpc. At necropsy, the main gross lesions in the challenge control group were pericardial effusion and swollen, friable, and yellow-stained livers accompanied by necrosis and bleeding (Figure 4a), which are consistent with reports in previous studies [27]. However, the chickens in groups I, II and IV, as well as those in the negative control group, remained healthy and active throughout the experimental period and there were no gross lesions at necropsy. In comparison, the chickens in group III showed mild depression and decreased appetites; after they had been euthanized, their livers were seen to be yellow and swollen, while pericardial effusion was not obvious (Figure 4a).

The vaccinated groups had complete protection except for group III (20 μg of penton base), so we used histopathology and immunohistochemistry (IHC) tests to detect whether the tissues were damaged in microscopic observation. According to the survival curve and viral load measurement in this study, we found that the FAdV-4 incidence rate peaked at 3 dpc. Therefore, we selected the tissues at 3 dpc for histopathology and IHC tests. The characteristic pathological changes in HHS were observed in the hearts, livers, and spleens of chickens in the challenged control group (Figure 4b). A part of the myocardial fiber with fractured and red blood cells can be observed in the myocardial interstitial space. The hepatic lobule structure disappeared, the hepatocyte exhibited nuclear fragmentation, and severe reduction and necrosis of lymphocytes were also observed. The organs of the chickens immunized with 20 μg of the penton base protein showed mild histopathological changes, such as an unclear hepatic lobule structure, a widened myocardial fiber gap, and some necrosis in the lymphocytes. Comparatively, no obvious histopathological lesions were observed in the tissue slices of groups I, II, IV, and the negative control.

An immunohistochemistry analysis detected many viral particles in the liver slices of chickens in the challenged control group and detected fewer viral particles in the heart and spleen slices, indicating that the chickens infected with FAdV-4 suffered serious infection and the most severely damaged tissue was the liver. A few viral particles were observed in the liver sections of the chickens vaccinated with 20 μg of the penton base protein. Other tissue sections tested negative for FAdV-4 (Figure 4c).

### 3.6. Determination of Minimum Immune Dose and Safety of Recombinant Fiber-2 Protein and Penton Base Protein

**Figure 5:**
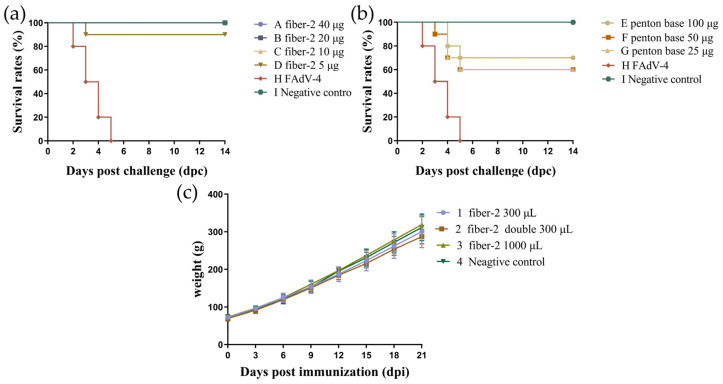
Determination of minimum immune dose of fiber-2 protein (**a**), penton base protein (**b**), and safety of oil adjuvants (**c**). Survival curves of all chickens in minimum immune dose of fiber-2 protein and penton base test following challenge with 2 × 10^6.5^ TCID_50_ highly pathogenic FAdV-4 JS strain at 21 dpi. The mortality rate was recorded for 14 dpc (**a**,**b**). Body weight curve of chickens subcutaneously injected with different volumes of fiber-2 subunit vaccine emulsified with Marcol™ 52 white oil at one site, and the body weight of all chickens were captured every 3 days until 21 dpi. *p* values were calculated with two-way ANOVA (**c**).

To investigate the minimum immune doses of recombinant fiber-2 protein and penton base protein, the chickens in groups A–D were inoculated with 40 μg, 20 μg, 10 μg, and 5 μg of fiber-2 protein, respectively, and the chickens in groups E–G were inoculated with 100 μg, 50 μg, and 25 μg of penton base protein. The results show that 40 μg, 20 μg, and 10 μg of recombinant fiber-2 protein could provide 100% (10/10) protection, and only 5 μg of fiber-2 protein provided 90% (9/10) protection (Figure 5a). In comparison, 100 μg of penton base proteins conferred 70% (7/10) protection, and 50 μg and 25 μg only confer 60% (6/10) protection (Figure 5b). The mortality rate of chickens in the challenge control group was 100%. Therefore, the minimum immune dose of fiber-2 protein that can provide complete protection is 10 μg/bird, and the minimum immune dose of penton base protein is 200 μg/bird.

Then, to confirm the safety of the fiber-2 subunit vaccine, purified recombinant fiber-2 proteins with a concentration of 0.1 mg/mL were emulsified with Marcol™ 52 white oil in a ratio of 1:2 (*v*/*v*). And the chickens in groups 1–3 were subcutaneously injected with different volumes of the above subunit vaccine at one site. We did not observe a serious inflammatory response where the vaccine was injected, and the vaccine was absorbed relatively well. More importantly, within 21 days after vaccination, the body weight of the chickens was not affected (Figure 5c).

## 4. Discussion

Various vaccine candidates against the highly pathogenic FAdV-4 have been developed, such as live attenuated vaccines [35,36], inactivated vaccines [35,37], and subunit vaccines [29,30,31,32,33,34]. However, live attenuated vaccines and inactivated vaccines are at risk of incomplete weakening or inactivation and reversion to virulence [3]. In addition, preparing inactivated vaccines is cumbersome and includes a high manufacturing cost [38]. In contrast, subunit vaccines are easier to obtain and much cheaper to produce [26], and related reports have shown that subunit vaccines can induce a higher level of antibodies than traditional oil emulsion inactivated vaccines [31]. 

The prokaryotic expression system has became the first choice for the preparation of subunit vaccines due to its high yield, low cost, and easy management. However, in previous studies, fiber-2 proteins expressed in prokaryotic expression systems were often found in the form of inclusion bodies [26,27,29,31]. Although the inclusion body form of the fiber-2 protein could also provide full protection against FAdV-4, refolding an inclusion body protein to restore biological activity is a complicated and cumbersome step with a low success rate [39,40], limiting the use of recombinant proteins in vaccine production. Therefore, to avoid the influence of complex refolding steps on the biological activity of protein, we achieved the soluble expressions of the fiber-2 and penton base proteins by reducing the culture temperature to 16 °C. Using a lower culture temperature will cause bacteria to grow at a slower rate, giving the protein sufficient time to fold so that it can be expressed in a soluble form. In addition, the use of Rosetta (DE3) competent cells, which provide a higher yield of proteins than BL21 (DE3) competent cells due to a plasmid named pRARE encoding rare codon tRNAs [41], cause fiber-2 proteins to reach a concentration of almost 1.5 mg/mL. Other research results have also shown that fiber-2 protein could be expressed as a soluble form in prokaryotic expression systems [30,42]. Strikingly, in our study, the yield of purified fiber-2 proteins obtained per liter LB medium almost reached 170 mg, which is much higher than other similar studies [30,34,42]. 

Adjuvants have a very important impact on the efficacy of vaccines. Based on this, almost all studies about subunit vaccines against FAdV-4 use expensive adjuvants to enhance immunogenicity. The immunogenicity of fiber-2 expressed in *E. coli* has been extensively researched and confirmed, which was emulsified with Sigma adjuvant [33], Freund’s complete adjuvant [31], Montanide ISA 71VG [30,32], and so on. However, these studies have ignored a very important point: adjuvants with good safety and low cost are more attractive for the industrial production of animal vaccines. Therefore, we developed a subunit vaccine formulated with cheap and widely used oil adjuvants. Lower immune doses can be found in recent reports, in which the minimum immune doses of fiber-2 protein are 5 µg/bird [32], 2.5 µg/bird [31], and 2 µg/bird [42], respectively. However, we did not use the same adjuvants that may include immune-enhancing additives such as mycobacterial cell wall components. This may be the reason the subunit vaccine we prepared requires a higher immune dose (10 µg/bird) to provide full protection. In addition, it is worth mentioning that although the titer of the antibody induced by a 20 µg penton base is higher than that of fiber-2 at 7 dpi, and basically the same as fiber-2 at 14 dpi and 21 dpi, it does not provide full protection. The reasons for this need to be further explored in subsequent experiments. 

The T-cell immune response plays a key role in suppressing virus replication [43,44]. Recent studies have shown that although the fiber-2 protein cannot induce neutralizing antibodies, it can still provide protective effects against FAdV-4 and suppress virus replication. This may rely on the local cellular immune response in target organs [45]. Our research results suggest that, although the viral loads in the tissues from chickens vaccinated with fiber-2 protein subunit vaccines were significantly lower than those in the challenged control group, the virus was still detected in the cloacal swabs from vaccinated chickens. For large-scale chicken farms, reducing virus shedding can effectively prevent pollution in the breeding environment, thereby reducing the risk of virus transmission. Therefore, multi-epitope subunit vaccines that could induce T-cell immune response may be the future development trend of subunit vaccines [44].

## 5. Conclusions

In summary, we developed a fiber-2 subunit vaccine that confers 100% protection at a low dose and has good industrialization potential. More importantly, for the first time, we have demonstrated the potential of using oil adjuvants (Marcol™ 52 white oil), which are widely used to produce inactivated vaccines, to produce subunit vaccines against the highly pathogenic FAdV-4.

## Figures and Tables

**Table 1:** The sequence of primers used in this study.

Primers Name	Sequence (5′-3′) ^b^	Produces Sizes (bp)
fiber 2-F	ggtcgcgatccgaattcATGCTCCGGGCCCCTAAAAGA	1440
fiber 2-R	gtggtggtggtgctcgagTTACGGGAGGGAGGCCGCTGG	
penton base-F	ggtcgcgatccgaattcATGTGGGGGTTGCAGCCGCCGA	1578
penton base-R	gtggtggtggtgctcgagTACTGCAAGGTCGCGGAACTC	
qORF14-F ^a^	AGTGTGTATGTGCGTTGGGTAG	136
qORF14-R ^a^	CATTGTCATAACGATGGTGTAG	

^a^ Primers of FAdV-4 ORF14 gene used in this study are from reference [8]. ^b^ Lowercase letters represent the sequences of pET-28a vector.

## Data Availability

All data that support this study are available from the corresponding author and Supplementary Materials upon reasonable request.

## Supplementary Materials

The following supporting information can be downloaded at: https://www.mdpi.com/article/10.3390/vaccines12030263/s1, Figure S1. SDS-PAGE analysis of soluble and pellet of *E. coli* expressing penton base and fiber-2 proteins. M, protein ladder. Lane 1, *E. coli* Rosetta (DE3) containing pET-28a with IPTG induction. Lane 2, total extracts of *E. coli* Rosetta (DE3) containing pET-28a-fiber-2. Lane 3, soluble extracts of *E. coli* Rosetta (DE3) containing pET-28a-fiber-2. Lane 4, purified recombinant fiber-2 protein. Lane 5, total extracts of *E. coli* Rosetta (DE3) containing pET-28a-penton base. Lane 6, soluble extracts of *E. coli* Rosetta (DE3) containing pET-28a-penton base. Lane 7, purified recombinant penton base protein. Figure S2. Western blot analysis of the recombinant proteins probed by anti-histamine monoclonal antibody. M, protein ladder. Lane pET28a, *E. coli* Rosetta (DE3) containing pET-28a with IPTG induction. Lane fiber-2, purified recombinant fiber-2 protein. Lane penton base, purified recombinant penton base protein. Figure S3. Western blot analysis of the recombinant proteins probed by anti-Fiber-2 monoclonal antibody. M, protein ladder. Lane pET28a, *E. coli* Rosetta (DE3) containing pET-28a with IPTG induction. Lane fiber-2, purified recombinant fiber-2 protein. Lane penton base, purified recombinant penton base protein. Figure S4. Western blot analysis of the penton base protein probed by anti-penton base positive chicken serum. M, protein ladder. Lane pET28a, *E. coli* Rosetta (DE3) containing pET-28a with IPTG induction. Lane fiber-2, purified recombinant fiber-2 protein. Lane penton base, purified recombinant penton base protein.
