# Supplementary material for: The Development of a Novel Fiber-2 Subunit Vaccine against Fowl Adenovirus Serotype 4 Formulated with Oil Adjuvants
Source: Vaccines (Basel). 2024 Mar 1;12(3):263. doi: 10.3390/vaccines12030263 (PMC10975688; doi:10.3390/vaccines12030263)
Supplement: Supplementary file 1 [file vaccines-12-00263-s001.zip › vaccines-2878781-supplementary.pdf]

Figure S1

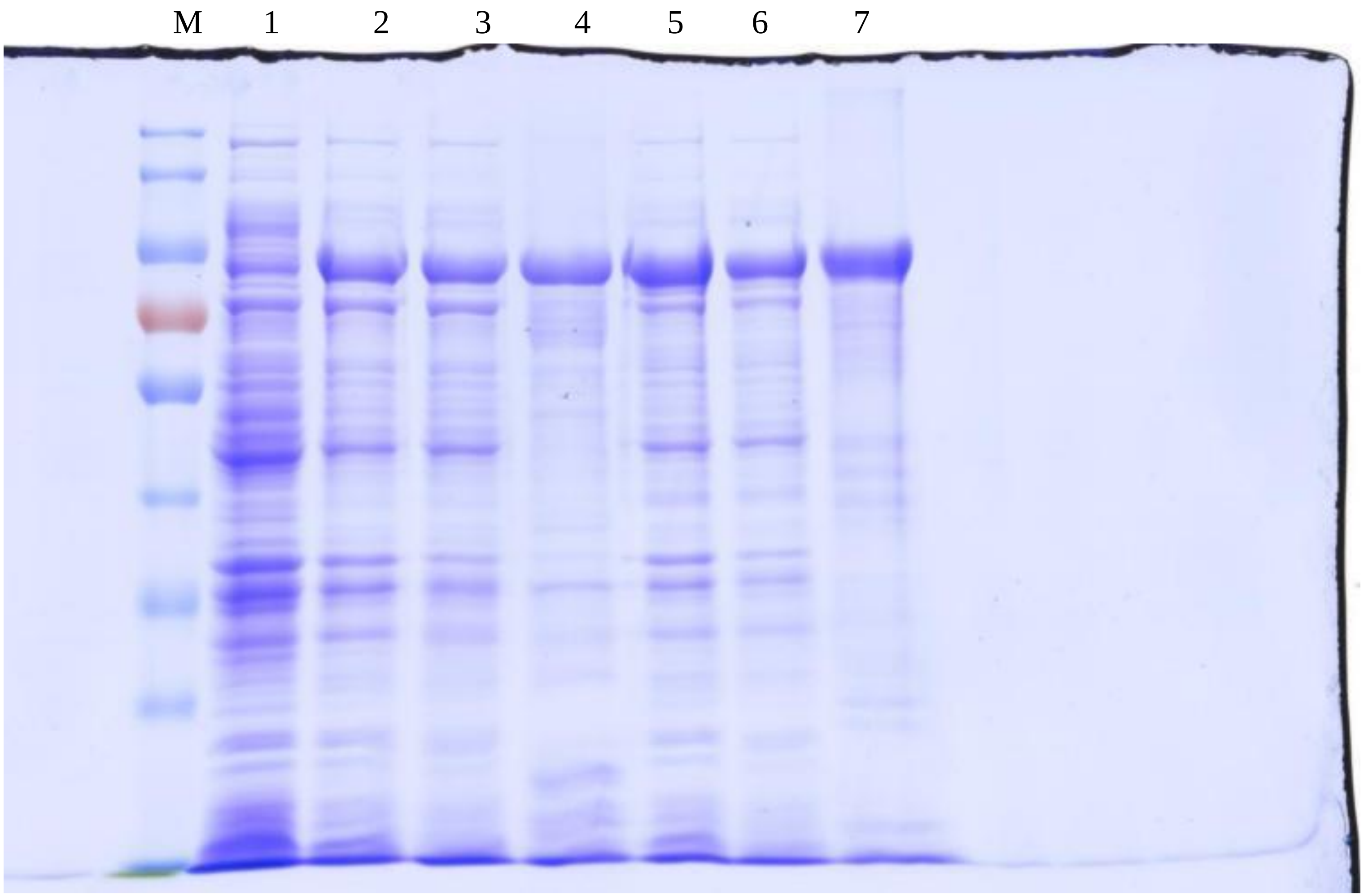

SDS-PAGE analysis of soluble and pellet of E. coli expressing penton base and fiber-2 proteins

M, protein ladder. Lane 1, E. coli Rosetta (DE3) containing pET-28a with IPTG induction. Lane 2, total extracts of E. coli Rosetta (DE3) containing pET-28a-fiber-2. Lane 3, soluble extracts of E. coli Rosetta (DE3) containing pET-28a-fiber-2. Lane 4 purified recombinant fiber-2 protein. Lane 5, total extracts of E. coli Rosetta (DE3) containing pET-28a-penton base. Lane 6, soluble extracts of E. coli Rosetta (DE3) containing pET-28a-penton base. Lane 7 purified recombinant penton base protein.

Figure S2

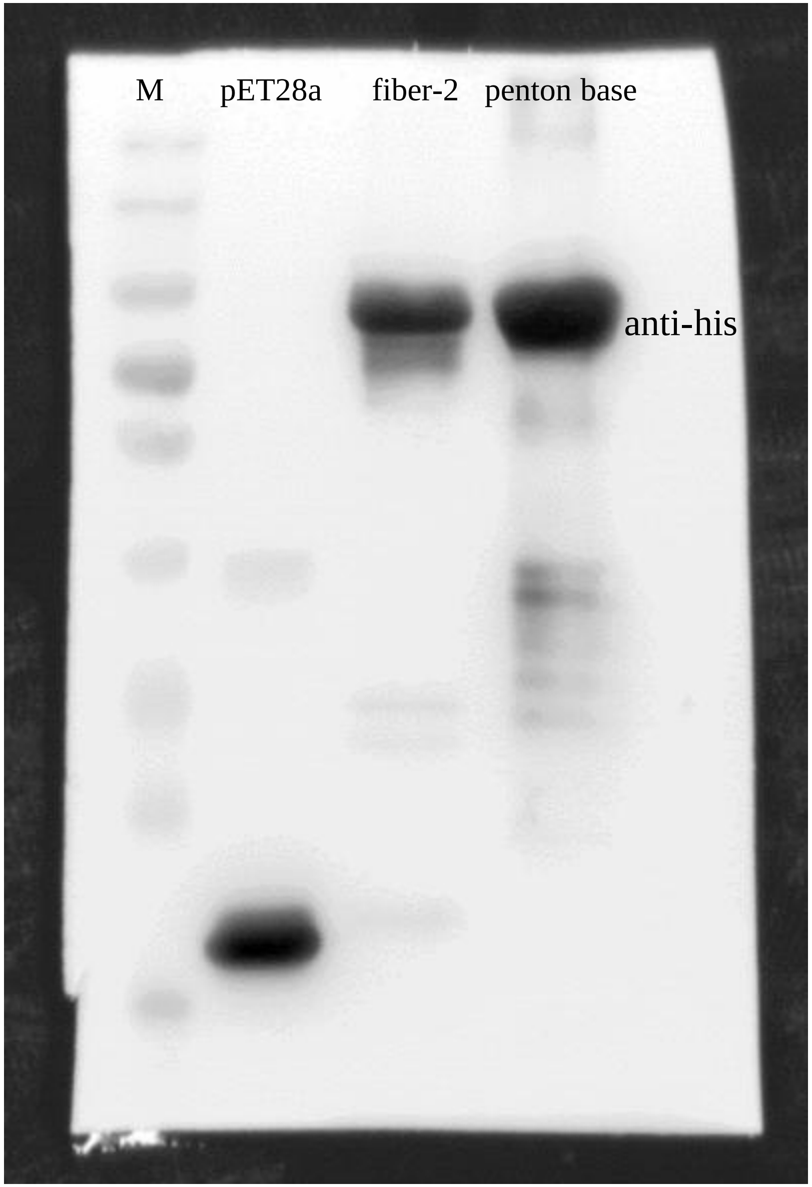

Western blot analysis of the recombinant proteins probed by anti-histamine monoclonal antibody
M, protein ladder. Lane pET28a, E. coli Rosetta (DE3) containing pET-28a with IPTG induction. Lane fiber-2, purified recombinant fiber-2 protein. Lane penton base, purified recombinant penton base protein.

Figure S3

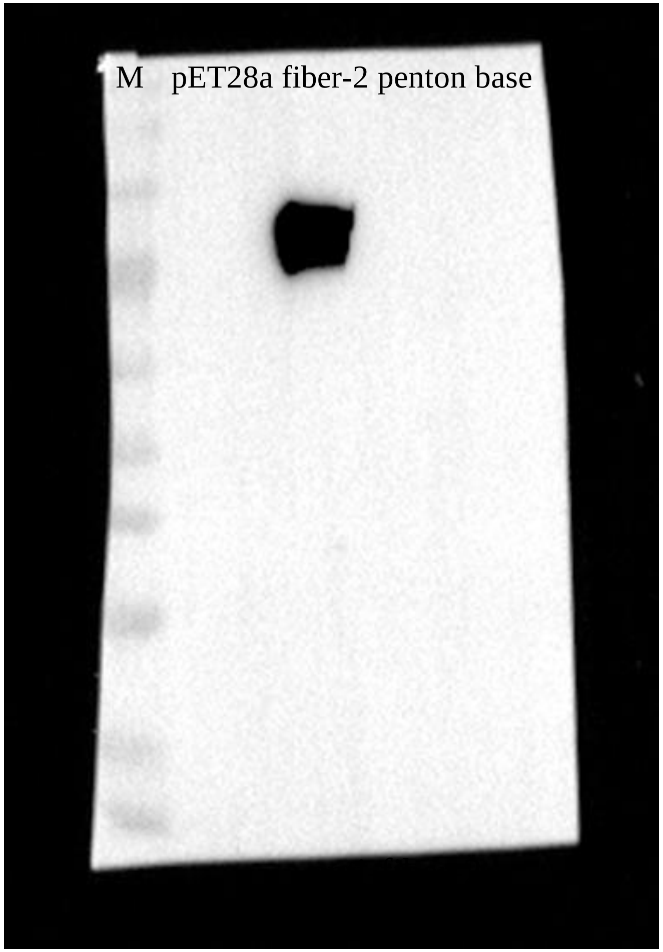

Western blot analysis of the recombinant proteins probed by anti-Fiber-2 monoclonal antibody
M, protein ladder. Lane pET28a, E. coli Rosetta (DE3) containing pET-28a with IPTG induction. Lane fiber-2, purified recombinant fiber-2 protein. Lane penton base, purified recombinant penton base protein.

Figure S4

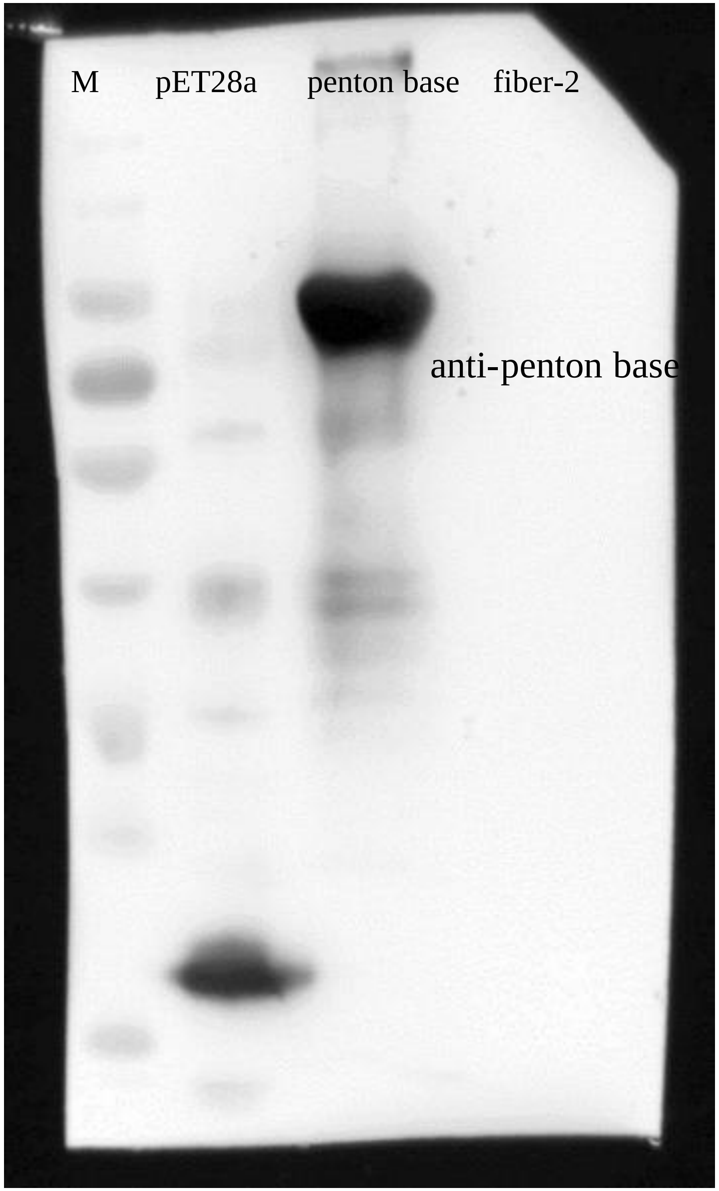

Western blot analysis of the penton base protein probed by chicken polyclonal antibodies against anti-penton base positive chicken serum

M, protein ladder. Lane pET28a, E. coli Rosetta (DE3) containing pET-28a with IPTG induction. Lane fiber-2, purified recombinant fiber-2 protein. Lane penton base, purified recombinant penton base protein.
